# ‘THEY ASKED ME TO JOIN; I JOINED:’ LESSONS FOR SENIOR CENTERS ON SUPPORTING PHYSICAL ACTIVITY

**DOI:** 10.1093/geroni/igac059.2881

**Published:** 2022-12-20

**Authors:** Sarah Chard, John Schumacher

**Affiliations:** UMBC, Baltimore, Maryland, United States; University of Maryland, Baltimore County, Baltimore, Maryland, United States

## Abstract

Among older adults, physical activity (PA) remains a foundation for maintaining and improving health. However, over 25% of adults aged ≥ 50 engage in no physical activity outside of work hours (CDC 2016). PA does correlate with senior center participation but little is known about the social factors that contribute to sustaining senior center PA routines. We report on findings from qualitative interviews conducted with Black older adults (Nf22) on the “story” of their senior center involvement, particularly their PA participation. Interviews were recorded and transcribed verbatim; co-authors independently conducted a line-by-line analysis to identify themes. They then reconciled and sorted themes. Four themes provided insights on participants’ PA engagement: 1) Retirement as opportunity; 2) Invitation and welcome; 3) Physician messaging; and 4) Sense of community. Participants strongly connected retirement with the opportunity to engage in regular PA; work and family obligations previously prevented PA in daily life. Participants also linked trying new PA programs to the senior center’s culture of extending one-to-one invitations. Physician messaging specifically “to exercise” provided additional validation for prioritizing senior center PA within daily schedules. Finally, a sense of community, of being missed if not attending, provided further incentive to sustain PA routines. These findings provide guidance and simple lessons that senior centers can employ to encourage participation in their PA programming. The themes may also inform the design of survey research examining patterns of PA among older adults.

